# Clinicopathological and prognostic features of HER2-null and HER2-low advanced breast cancer treated with eribulin or capecitabine

**DOI:** 10.1007/s12282-024-01617-y

**Published:** 2024-08-14

**Authors:** Rui Kitadai, Tatsunori Shimoi, Shu Yazaki, Hitomi Sumiyoshi Okuma, Mai Hoshino, Munehiro Ito, Ayumi Saito, Shosuke Kita, Yuki Kojima, Tadaaki Nishikawa, Kazuki Sudo, Emi Noguchi, Yasuhiro Fujiwara, Masayuki Yoshida, Kan Yonemori

**Affiliations:** 1 Department of Medical Oncology, National Cancer Center Hospital, 5-1-1 Tsukiji, Chuo-ku, Tokyo, 104-0045 Japan; 2 Department of Diagnostic Pathology, National Cancer Center Hospital, Tokyo, Japan

**Keywords:** HER2-low, Advanced breast cancer, Eribulin, Capecitabine, Overall survival

## Abstract

**Background:**

HER2-low populations constitute a heterogeneous group, and the cytotoxic anticancer agent efficacy based on HER2 status remains unclear. This study evaluated the clinicopathological features and outcomes of patients with advanced breast cancer showing HER2-low expression treated with eribulin or capecitabine, two treatment options after anthracycline and taxane treatment.

**Methods:**

We retrospectively evaluated patients who were treated with eribulin or capecitabine between 2011 and 2015. HER2 status was evaluated according to the ASCO/CAP guidelines.

**Results:**

No significant difference was observed in overall survival (OS; eribulin: hazard ratio [HR], 0.66; 95% CI 0.40–1.10; capecitabine: HR, 0.76; 95% CI 0.45–1.30) or progression-free survival (PFS; eribulin: HR, 1.13; 95% CI 0.72–1.78; capecitabine: HR, 0.90; 95% CI 0.56–1.44) between patients receiving eribulin (HER2-null: 35, HER2-low: 44) and those receiving capecitabine (HER2-null: 41, HER2-low: 33). Subgroup analysis revealed no significant differences in OS between the two groups in the hormone-positive and -negative populations for eribulin and capecitabine. HER2-null and HER2-low patients showed objective response rates (ORRs) of 22.5% and 9.1% (p = 0.09) overall, and 32.0% and 10.5% (p = 0.03), respectively, in hormone-positive cases among eribulin-treated patients. No response was observed in hormone-negative patients. Capecitabine treatment in HER2-null and HER2-low patients had overall ORRs of 26.8% and 15.2% (p = 0.23), respectively, with 27.3% and 16.1% (p = 0.28) for hormone-positive cases; and 25.0% and 0% (p = 1.0), respectively, for hormone-negative cases.

**Conclusions:**

Eribulin and capecitabine sensitivity may vary based on HER2 expression in patients with HER2-low and HER2-null breast cancer. Prognosis was similar between the HER2-low and the HER2-null groups.

## Introduction

Breast cancer is a malignant disease encompassing various molecular subtypes. In clinical practice, it is divided into subtypes based on hormone receptors [estrogen receptor (ER) and progesterone receptor (PgR)] and human epidermal growth factor receptor 2 (HER2) expression. HER2 is a tyrosine kinase receptor involved in cell proliferation, migration, invasion, and survival [1]. It is a negative prognostic factor [2], and its overexpression is observed in approximately 15%–20% of patients with breast cancer [3]. The development of HER2-targeted treatments has revolutionized the natural course of HER2-positive breast cancer, effectively prolonging the survival of patients with advanced-stage breast cancer. The treatment landscape has become more diverse with the emergence of trastuzumab and the subsequent development of other HER2-targeted agents [4], enabling different treatment sequences for each clinical setting.

Recently, HER2-low expression defined as HER2 1 + , HER2 2 + , and HER2 in situ hybridization (ISH)-negative, has been suggested to have distinct prognostic implications [5–7]. HER2-low populations constitute a heterogeneous group, including HR-positive and -negative breast cancers that vary in prognosis and response to treatments. Targeted therapies have been developed, given that approximately 60% of HER2-negative metastatic breast cancers are low-HER2 [8, 9]. Among these treatments, trastuzumab deruxtecan (T-DXd) is an anti-HER2 drug with remarkable efficacy against metastatic HER2-low breast cancer (DESTINY-Breast04) [10]. However, it is important to investigate whether HER2-low status affects prognosis and sensitivity to systemic treatment to develop a treatment strategy for individuals across different clinical settings.

The efficacy of anticancer agents other than trastuzumab deruxtecan was collectively studied as a treatment of choice in the DESTINY-Breast04 trial [10]. However, the relationship between the efficacy of cytotoxic anticancer agents studied as a treatment of physician’s choice (TPS), and the HER2-low status has not been fully examined. Furthermore, eribulin and capecitabine appear to be the major drugs used following T-DXd based on the DB04, 301 [11], and EMBRACE [12] trials. Therefore, the present study focused on patients with metastatic or recurrent breast cancer treated with eribulin or capecitabine, both of which are treatment options following anthracycline and taxane treatment. We compared the clinicopathological features and prognosis of HER2-low and HER2-null patients. The results of this study provide valuable insights into the management and outcomes of the HER2-low population by analyzing a specific population of patients with advanced breast cancer.

## Materials and methods

### Study population

We initially included patients with metastatic or recurrent breast cancer who were treated with eribulin between 2011 and 2015 or with capecitabine between 2007 and 2015 at the National Cancer Center Hospital, Tokyo, Japan. Some patients received both eribulin and capecitabine treatments. We selected patients who were histologically proven to be HER2 0, HER2 1 + , or HER2 2 + plus HER2-ISH-negative. HER2 0 was defined as HER2-null, and HER2 1 + and HER2 2 + plus HER2-ISH negative were defined as HER2-low. For cases with specimens from both primary and metastatic lesions, the HER2 evaluation of the metastatic lesion was prioritized. For cases with only primary lesion specimens, the evaluation was based on the primary lesion. Pathologists evaluated the specimens and determined HER2 status according to the American Society of Clinical Oncology/College of American Pathologists guidelines at the time of diagnosis [13, 14]. HER2 protein was detected using immunohistochemical (IHC) analysis of formalin-fixed paraffin-embedded (FFPE) tissue specimens using Dako HercepTest II (DakoCytomation, Glostrup, Denmark), strictly following the manufacturer’s guidelines. For IHC 2 + cases, HER2 fluorescence in situ hybridization (FISH) was performed using PathVysion (Abbott Molecular Inc., Des Plaines, Illinois, USA). The pathological diagnoses were confirmed by at least two board-certified pathologists at our hospital. The patients received eribulin (1.4 mg/m^2^ on days 1 and 8 of a 21-day cycle) intravenously or capecitabine (either 2500 mg/m^2^/day for 14 days every 21 days or 1650 mg/m^2^/day for 21 days every 28 days) orally. Treatment was continued until documented or clinical disease progression, unacceptable toxicity, deterioration of the general condition, or patient refusal.

This study was approved by the Institutional Review Board of the National Cancer Center (Tokyo, Japan) [No. 2014-092]. This study was conducted in accordance with the principles of the Declaration of Helsinki. The requirement for informed consent was waived by the Institutional Review Board owing to the retrospective nature of the study.

### Data collection

Clinical data regarding age at diagnosis, sex, Eastern Cooperative Oncology Group Performance Status (ECOG PS), estrogen receptor (ER) status, progesterone receptor (PgR) status, histological grade, presence of visceral metastases, details of chemotherapy treatment, previous surgery, previous endocrine therapy, and previous neoadjuvant and adjuvant therapies were collected from medical records. Negative ER and PgR statuses were defined as < 1%, according to the American Society of Clinical Oncology/College of American Pathologists guidelines [13, 14]. Clinical responses were evaluated in patients with measurable lesions, according to the Response Evaluation Criteria in Solid Tumors (version 1.1) [15]. Overall survival (OS) was defined from the first day of chemotherapy until death or the date of last follow-up, while progression-free survival (PFS) was defined as the period from the first day of chemotherapy until disease progression or death prior to disease progression. The data cutoff date was May 31, 2023.

### Statistical analysis

Continuous variables were compared using the* t*-test for normally distributed data and the Mann–Whitney U test for non-normally distributed data, whereas categorical variables were compared using Fisher’s exact test. We compared PFS and OS between HER2-low and HER2-null patients and examined the factors that could affect prognosis. The Kaplan–Meier method was used to estimate OS and PFS, and survival curves were compared using the log-rank test. Cox proportional hazard models were used to evaluate the risk factors. All p-values were based on two-sided tests, with p < 0.05 considered statistically significant. Statistical analyses were conducted using SPSS (IBM Corp. Released 2021. IBM SPSS Statistics for Windows, Version 28.0. Armonk, NY: IBM Cor).

## Results

### Patient and clinical characteristics

A total of 91 and 87 patients with metastatic breast cancer received eribulin and capecitabine, respectively. After selecting patients with HER2-null and HER2-low status, 79 and 74 patients were retained in the analysis, respectively. The baseline characteristics of the patients are shown in Table 1. In the eribulin treatment group, HER2 evaluation was performed based on metastatic lesions in 27 cases and primary lesions in 52 cases. In the capecitabine treatment group, the evaluations were performed based on metastatic lesions in 30 cases and primary lesions in 44 cases.

**Table 1:** Baseline characteristics of patients in the HER2-null and HER2-low groups

Characteristics	Eriburin			Capecitabine		
	HER2-null (n = 35)	HER2-low (n = 44)	*p*-value	HER2-null (n = 41)	HER2-low (n = 33)	*p*-value
Median age, years (range)	54 (30–76)	55 (33–70)	1.0	59 (36–76)	56 (38–69)	0.45
ECOG PS, n (%)						
0	19 (54.3)	30 (68.2)	0.21	23 (56.1)	18 (54.6)	0.53
1	16 (45.7)	14 (31.8)		18 (43.9)	14 (42.4)	
2	0 (0.0)	0 (0.0)		0 (0.0)	1 (3.0)	
HER2 status, n (%)						
IHC 0	35 (100)	–	–	41 (100)	–	–
IHC 1 +	–	36 (81.8)		–	28 (84.8)	
IHC 2 + , ISH-negative	–	8 (18.2)		–	5 (15.2)	
HR, n (%)						
Positive	25 (71.4)	38 (86.4)	0.10	33 (80.5)	31 (93.9)	0.09
Negative	10 (28.6)	6 (13.6)		8 (19.5)	2 (6.1)	
Histological grade, n (%)						
1	1 (2.9)	0 (0.0)	0.48	2 (4.9)	0 (0.0)	0.035
2	16 (45.7)	22 (50.0)		13 (31.7)	23 (69.7)	
3	13 (37.1)	20 (45.5)		16 (39.0)	9 (27.3)	
NA	5 (14.3)	2 (4.5)		10 (24.4)	1 (3.0)	
Visceral metastases, n (%)						
No	12 (34.3)	6 (13.6)	0.03	13 (31.7)	6 (18.2)	0.19
Yes	23 (65.7)	38 (86.4)		28 (68.3)	27 (81.8)	
Median number of chemotherapy lines for metastatic disease, (range)	3 (1–4)	3 (1–4)	0.90	2 (1–3)	2 (1–3)	0.23
Treatment lines of chemotherapy for metastatic disease, n (%)						
1	3 (8.6)	2 (4.5)	0.76	11 (26.8)	11 (33.3)	0.36
2	11 (31.4)	15 (34.1)		18 (43.9)	17 (51.5)	
≥ 3	21 (60.0)	27 (61.4)		12 (29.3)	5 (15.2)	
Prior primary tumor resection, n (%)						
Yes	34 (97.1)	37 (84.1)	0.06	38 (92.7)	29 (87.9)	0.70
No	1 (2.9)	7 (15.9)		3 (7.3)	4 (12.1)	
Previous endocrine therapy, n (%)						
Yes	24 (68.6)	30 (68.2)	1.0	36 (87.8)	30 (90.9)	0.73
No	11 (31.4)	14 (31.8)		5 (12.2)	3 (9.1)	
Neo adjuvant/adjuvant chemotherapy, n (%)						
Yes	31 (88.6)	33 (75.0)	0.13	34 (82.9)	26 (78.8)	0.65
No	4 (11.4)	11 (25.0)		7 (17.1)	7 (21.2)	

*ECOG PS* Eastern cooperative oncology group performance status, *HER 2* human epidermal growth factor receptor 2, *HR* hormone receptor, *IHC* immunohistochemistry, *ISH* in situ hybridization, *NA* not available

Among the patients who received eribulin, 35 were HER2-null and 44 were HER2-low. The median ages were 54 years (range 30–76 years) and 55 years (range 33–70 years) in the HER2-null and HER2-low groups, respectively. Nineteen (54.3%) patients and 30 (68.2%) patients were performance status (PS) 0 in the HER2-null and HER2-low groups, respectively. Of the 44 HER2-low patients, 36 (81.8%) were IHC 1 + and 8 (18.2%) were IHC2 + plus ISH-negative. Twenty-five (71.4%) patients and 38 (86.4%) patients were hormone-positive in the HER2-null and HER2-low groups, respectively. Histological grades of 1, 2, 3, and NA accounted for 1 (2.9%), 16 (45.7%), 13 (37.1%), and 5 (14.3%) patients in the HER2-null group, and 0 (0%), 22 (50.0%), 20 (45.5%), and 2 (4.5%) patients in the HER2-low group, respectively. The HER2-null group had fewer patients with visceral metastases than the HER2-low group [23 (65.7%) vs. 38 (86.4%), p = 0.03]. The median number of chemotherapy lines for metastatic disease was three in both groups. Most patients [HER2-null group: 24 (97.1%); HER2-low group: 37 (84.1%)] had undergone prior primary tumor resection, and 31 (88.6%) in the HER2-null group and 33 (75.0%) in the HER2-low group received neoadjuvant or adjuvant chemotherapy.

Among patients who received capecitabine, 41 patients were HER2-null and 33 were HER2-low. The median ages were 59 (range 36–76 years) and 56 (range 38–69 years) in the HER2-null and HER2-low groups, respectively. Twenty-three (56.1%) patients and 18 (54.6%) patients were PS 0 in the HER2-null and HER2-low groups, respectively. Among the 33 HER2-low patients, 28 (84.8%) were IHC 1 + and 5 (15.2%) were IHC2 + plus ISH-negative. Thirty-three (80.5%) patients and 31 (93.9%) patients were hormone-positive in the HER2-null and HER2-low groups, respectively. In the HER2-null group, histological grades of 1, 2, 3, and NA accounted for 2 (4.9%), 13 (31.7%), 16 (39.0%), and 10 (24.4%) patients, respectively; in the HER2-low group, these grades accounted for 0 (0%), 23 (69.7%), 9 (27.1%), and 1 (3.0%) patients, respectively, which were significantly different (p = 0.035) compared to the HER2-null group. In the HER2-null and HER2-low groups, 28 (68.3%) and 27 (81.8%) of patients had visceral metastases, respectively. The median number of chemotherapy lines for metastatic disease was 2 in both groups. Most patients [HER2-null group: 38 (92.7%); HER2-low group: 29 (87.9%)] had a prior primary tumor resection; 34 (82.9%) in the HER2-null group and 26 (78.8%) in the HER2-low group received neoadjuvant or adjuvant chemotherapy.

### Treatment outcomes

**Fig. 1:**
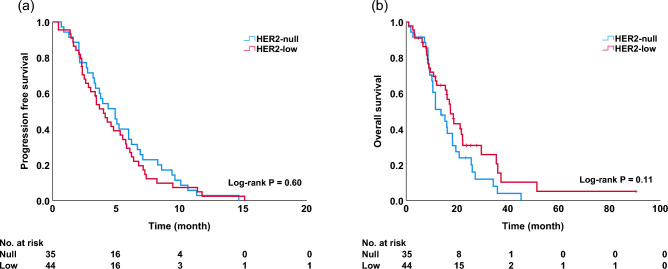
Progression-free survival (**a**) and overall survival (**b**) among patients treated with eribulin in the HER2-null and HER2-low groups

**Fig. 2:**
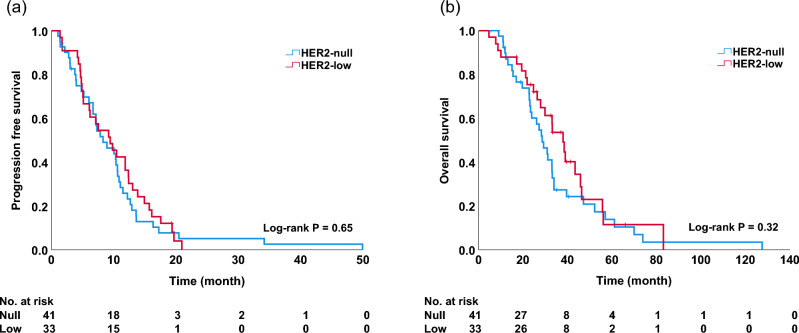
Progression-free survival (**a**) and overall survival (**b**) among patients treated with capecitabine in the HER2-null and HER2-low groups

**Fig. 3:**
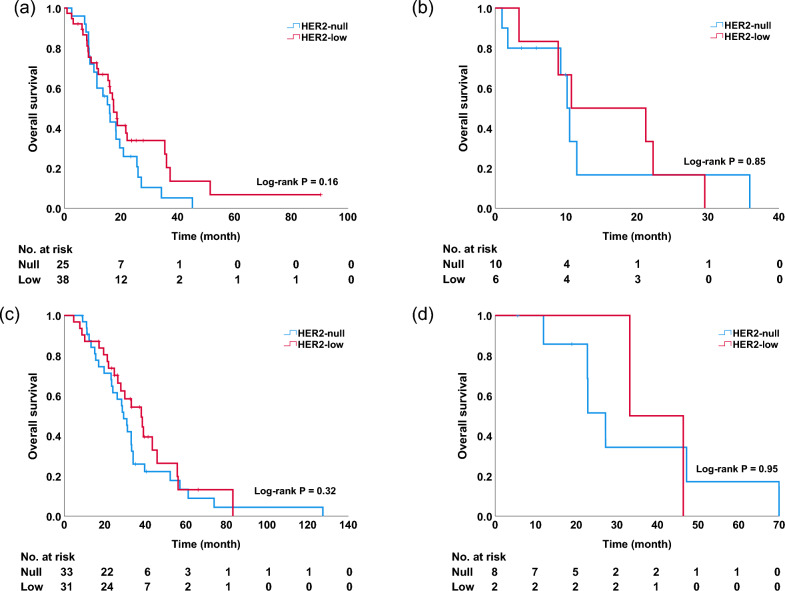
Overall survival among patients treated with eribulin or capecitabine in the HER2-null and HER2-low groups, according to the hormone receptor status: **a** hormone receptor-positive patients treated with eribulin, **b** hormone receptor-negative patients treated with eribulin, **c** hormone receptor-positive patients treated with capecitabine, and **d** hormone receptor-negative patients treated with capecitabine

Among patients receiving eribulin, there was no significant difference in OS [hazard ratio (HR), 0.66; 95% confidence interval (CI) 0.40–1.10] and PFS (HR, 1.13; 95% CI 0.72–1.78) between patients in the HER2-null and HER2-low groups [median PFS (mPFS): 4.9 vs. 4.0 months, p = 0.60, Fig. 1a; median OS (mOS): 13.6 vs. 17.4 months, p = 0.11, Fig. 1b). Univariate and multivariate analyses did not reveal any significant differences in OS (univariate, p = 0.11; multivariate, p = 0.59; Table 2). There was no significant difference in OS among patients receiving capecitabine, (HR, 0.76; 95% CI 0.45–1.30) or in PFS (HR, 0.90; 95% CI 0.56–1.44) between patients in the HER2-null and HER2-low groups (mPFS: 8.3 vs. 9.4 months, p = 0.65, Fig. 2a; mOS: 28.6 vs. 38.0 months, p = 0.32, Fig. 2b). Univariate and multivariate analyses did not reveal any significant differences in OS (univariate, p = 0.32; multivariate, p = 0.08; Table 3). A subgroup analysis based on hormonal status is shown in Fig. 3. There was no significant difference in OS between patients in the HER2-null and HER2-low groups in the hormone-positive population receiving eribulin (mOS: 16.0 vs. 17.4 months; p = 0.16) and the hormone-negative population (mOS: 10.1 vs. 10.7 months; p = 0.85) (Fig. 3a, b). In patients receiving capecitabine, there was no significant difference in OS between the two groups in both hormone-positive (mOS: 29.2 vs. 38.0 months; p = 0.32) and negative (mOS: 27.2 vs. 33.2 months; p = 0.95) populations (Fig. 3c, d).

**Table 2:** Univariate and multivariate analyses of overall survival in patients receiving eribulin

	Patient number	Univariate		Multivariate	
		HR (95% CI)	*p*-value	HR (95% CI)	*p*-value
HER2 status					
Null	35	1	–	1	–
Low	44	0.66 (0.40–1.10)	0.11	0.84 (0.45–1.59)	0.59
Age					
≤ 56	41	1	–	–	–
> 56	38	0.89 (0.54–1.49)	0.66	–	–
ECOG PS					
0	49	1	–	1	–
1	30	1.35 (0.80–2.27)	0.27	1.79 (0.98–3.27)	0.058
HR status					
Negative	16	1	–	1	–
Positive	63	0.63 (0.34–1.17)	0.14	0.59 (0.28–1.25)	0.17
Histological grade					
1	1	1	–	1	–
2	38	0.73 (0.98–5.40)	0.75	0.71 (0.09–5.71)	0.75
3	33	0.59 (0.34–1.03)	0.06	1.04 (0.12–8.69)	0.97
Visceral metastasis					
No	18	1	–	1	–
Yes	61	0.73 (0.41–1.29)	0.28	0.73 (0.34–1.58)	0.42
Treatment line					
< 3	31	1	–	1	–
≥ 3	48	0.83 (0.50–1.39)	0.48	1.07 (0.59–1.97)	0.82

*HER 2* Human epidermal growth factor receptor 2, *ECOG PS* Eastern cooperative oncology group performance status, *HR* hormone receptor

**Table 3:** Univariate and multivariate analyses of overall survival in patients receiving capecitabine

	Patient number	Univariate		Multivariate	
		HR (95% CI)	*p*-value	HR (95% CI)	*p*-value
HER2 status					
Null	41	1	–	1	–
Low	33	0.76 (0.45–1.30)	0.32	0.56 (0.30–1.06)	0.08
Age					
≤ 58	37	1	–	–	–
> 58	37	0.87 (0.52–1.46)	0.59	–	–
ECOG PS					
0	41	1	–	1	–
1	32	1.69 (0.96–2.96)	0.07	1.54 (0.83–2.85)	0.17
2	1	0.69 (0.09–5.11)	0.72	NA	NA
HR status					
Negative	10	1	–	1	–
Positive	64	0.93 (0.44–1.98)	0.86	0.78 (0.27–2.26)	0.65
Histological grade					
1	2	1	–	1	–
2	36	0.35 (0.08–1.53)	0.16	0.44 (0.94–2.07)	0.30
3	25	0.83 (0.20–3.60)	0.81	0.90 (0.20–4.04)	0.89
Visceral metastasis					
No	19	1	–	1	–
Yes	55	0.92 (0.52–1.61)	0.76	1.05 (0.48–2.27)	0.91
Treatment line					
< 3	57	1	–	1	–
≥ 3	17	1.16 (0.63–2.14)	0.63	1.14 (0.51–2.56)	0.75

*HER 2* Human epidermal growth factor receptor 2, *ECOG PS* Eastern cooperative oncology group performance status, *HR* hormone receptor

Table 4 shows the objective response rate (ORR) and disease control rate (DCR) of patients in the HER2-null and HER2-low groups among all patients, the hormone-positive population, and the hormone-negative population. Among patients treated with eribulin, those with HER2-null and HER2-low status exhibited ORRs of 22.5% and 9.1% (p = 0.09) in all patients and 32.0% and 10.5% (p = 0.03) in hormone-positive patients, respectively. The hormone-negative patients did not exhibit any response. Among patients treated with capecitabine, those with HER2-null and HER2-low status exhibited ORRs of 26.8% and 15.2% (p = 0.23) in all patients, while ORRs of 27.3% and 16.1% (p = 0.28) were observed in hormone-positive patients, respectively and 25.0% and 0% (p = 1.0), in hormone-negative patients, respectively. Although a significant difference was only observed in the hormone-positive population of patients receiving eribulin, the ORR was higher in the HER2-null group than in the HER2-low group. There were no significant differences in the DCR between the HER2-null and HER2-low groups.

**Table 4:** Objective response and disease control rate in HER2-null and HER2-low patients

	Total			HR-positive			HR negative		
	HER2-null	HER2-low	*p*-value	HER2-null	HER2-low	*p*-value	HER2-null	HER2-low	*p*-value
Eriburin, *n* (%)									
ORR	8 (22.9)	4 (9.1)	0.09	8 (32.0)	4 (10.5)	0.03	0 (0)	0 (0)	NA
DCR	23 (65.7)	25 (56.8)	0.42	19 (76.0)	22 (57.9)	0.14	4 (40.0)	3 (50.0)	1.0
Capecitabine, *n* (%)									
ORR	11 (26.8)	5 (15.2)	0.23	9 (27.3)	5 (16.1)	0.28	2 (25.0)	0 (0)	1.0
DCR	31 (75.6)	29 (87.9)	0.18	27 (81.8)	27 (87.1)	0.73	4 (50.0)	2 (100)	0.47

*HER 2* Human epidermal growth factor receptor 2, *HR* hormone receptor, *ORR* overall response rate, *DCR* disease control rate

## Discussion

We evaluated the efficacy of two cytotoxic anticancer agents against recurrent metastatic HER2-low breast cancer. Drug sensitivity may vary based on HER2 expression, although prognosis may not differ between the HER2-low and HER2-null groups. Several studies have investigated the response to treatment and the prognosis of HER2-low and HER2-null breast cancers. Notably, the effectiveness of chemotherapy indicated by a pathological complete response (pCR) appears to be significantly lower in HER2-low breast cancer than in HER2-null breast cancer. A comprehensive pooled analysis of four prospective neoadjuvant clinical trials involving 2310 patients showed a significantly lower pCR rate in HER2-low breast cancer than in HER2-null breast cancer (29.2% vs. 39.0%, p = 0.0002). This trend was more pronounced in the hormone receptor-positive subgroup (17.5% vs. 23.6%, p = 0.024) than in the hormone receptor-negative subgroup (50.1% vs. 48.0%, p = 0.21) [16]. A systematic review and meta-analysis of 42 studies confirmed the above findings and reported a lower rate of pCR in HER2-low tumors (odds ratio [OR], 0.74; 95% CI 0.62–0.88; p = 0.001), particularly in the hormone receptor-positive subgroup (OR, 0.77; 95% CI 0.65–0.90; p = 0.001) [6]. Moreover, a large-scale retrospective cohort analysis demonstrated the association with a slightly lower rate of pCR in ERBB2-low status patients (adjusted OR 0.89, 95% CI 0.86–0.92, p < 0.001) [17]. These results suggest that the efficacy of chemotherapy may differ based on HER2 status, and prognosis appears to differ between HER2-low and HER2-null breast cancers. A meta-analysis presented a significant difference in terms of OS in favor of patients with HER2-low breast cancer (HR, 0.94; 95% CI 0.89–0.98; p = 0.008), regardless of hormone status [6]. In contrast, no significant difference was found in terms of PFS after first-line treatment between HER2-low and HER2-null tumors [6]. In addition, a comprehensive pooled analysis [16] revealed that patients with HER2-low-positive tumors have significantly longer survival than those with HER2-zero tumors in the early setting, with 3-year overall rates of 91.6% and 85.8%, respectively (p = 0.0016). This difference was also observed in the hormone receptor-negative subgroups.

In this study, there were no significant differences in OS or PFS between the HER2-low and HER2-null groups in either the eribulin or capecitabine treatment groups. However, ORR tended to be poorer in the HER2-low group than in the null group, especially in the HR-positive subgroup of eribulin-treated patients, which is consistent with a previous report [16]. This molecular mechanism may explain the differences in chemotherapy between the HER2-low and HER2-null groups. Previous investigations have identified an overrepresentation of the luminal A molecular subtype, a subtype known for lower rates of pCR while maintaining a good prognosis, in HER2-low cancers [8, 18], which explains the poor response to chemotherapy in the HER2-low group. In addition, the HER2-null HR-positive group showed a notably high ORR among patients who received eribulin. Subgroup analysis of a phase III study of eribulin vs. capecitabine in patients with locally advanced or metastatic breast cancer [11] showed that patients receiving eribulin have better PFS in HER2-null patients than those who received capecitabine; however, the chemotherapy response according to the hormone status was unclear.

The proportion of HER2-low HR-positive breast cancers in this study did not significantly differ from that reported in other studies. One reason for the difference in OS between our study and previous reports may be that the HER2 evaluation samples were mixed for primary and metastatic diseases. HER2 status variability in metastasis and heterogeneity within organs is known, with a report of potentially significant discordance in which the HER2 status in 44% of breast cancers shifted from HER2-negative to HER2-low; conversely, 22% exhibit the opposite trend [19]. This previous study investigated the effect of HER2 expression on metastasis after recurrence. Biopsies were performed to reassess the subtype in cases where biopsy of metastases was possible after recurrence. However, in instances where biopsy after recurrence was not feasible, biopsies of the primary tumor were utilized. The meta-analysis mentioned earlier included a combination of studies that assessed HER2 expression in the primary tumor and those that examined metastases when biopsy was feasible, or in the primary tumor when biopsy of metastases was challenging [6], which we believe is an important factor that may have an impact on prognosis. However, there are insufficient data evaluating the response to chemotherapy among patients with HER2-low and HER2-null metastatic breast cancer. No significant differences in prognosis were reported based on HER2 status in a retrospective analysis that evaluated the prognosis of patients who received initial and second-line chemotherapy for HR-positive HER2-low or negative patients [20].

This study has some limitations. First, it was a retrospective study conducted at a single institution with a small sample size, which may affect the accuracy of the efficacy and prognosis evaluations, especially in the analysis according to hormone receptor status. Second, the patients were enrolled over a considerable time span, and the treatments did not align with modern standards, such as T-DXd for HER2-low patients and sacituzumab govitecan for HR + HER2 negative disease, which could affect prognosis evaluations. However, this study provides valuable insights, considering the paucity of data on the efficacy of multiple cytotoxic anticancer agents against HER2-low and HER2-null metastatic recurrent breast cancer.

## Conclusion

The results of this study suggest that drug sensitivity to eribulin and capecitabine may vary based on HER2 expression among patients with HER2-low and HER2-null breast cancers; however, there was no difference in prognosis between the two groups.

## Data Availability

The data underlying this article are available from the corresponding author upon reasonable request.
